# Optical Glucose Sensor Using Pressure Sensitive Paint

**DOI:** 10.3390/s21134474

**Published:** 2021-06-30

**Authors:** Jongwon Park

**Affiliations:** Department of Biomedical Engineering, Kyungil University, Gyeongsan 38428, Korea; jpark3@kiu.ac.kr; Tel.: +82-53-600-5721

**Keywords:** optical glucose sensor, optical oxygen sensor, pressure sensitive paint, photometry

## Abstract

A glucose sensor is used as an essential tool for diagnosing and treating diabetic patients and controlling processes during cell culture. Since the development of an electrochemical-based glucose sensor, an optical glucose sensor has been devised to overcome its shortcomings, but this also poses a problem because it requires a complicated manufacturing process. This study aimed to develop an optical glucose sensor film that could be fabricated with a simple process using commercial pressure sensitive paints. The sensor manufacturing technology developed in this work could simplify the complex production process of the existing electrochemical or optical glucose sensors. In addition, a photometric method for glucose concentration analysis was developed using the color image of the sensor. By developing this sensor and analysis technology, the basis for glucose measurement was established that enables two-dimensional, online, and continuous measurement. The proposed sensor showed good linearity at 0–4 mM glucose in an aqueous sample solution, its limit of detection was 0.37 mM, and the response time was 2 min.

## 1. Introduction

Monitoring of blood glucose is a very important procedure in clinical analysis for diabetic patients. By monitoring their own blood sugar, diabetic patients can check the tendency of glucose control in their body, factors changing the glucose levels, and effects of activities, diet, and drugs. As a result, it can be used to maximize the effect of diabetes treatment [1].

In addition, glucose is a major source of energy for cell growth. The medium used for cell cultures includes a carbon source, energy source, nitrogen source, inorganic salts, and trace elements. In addition to these, vitamins, growth factors, and buffers are added to animal cell cultures. In order to optimize the environment for the purpose of cell culture, it is necessary to adjust it by measuring the concentration of various substances, such as glucose and oxygen, in real time, in addition to maintaining the temperature and pH [2].

Until now, many methods for measuring glucose in blood or in various samples, such as a medium for cell culture, have been suggested, and commercial products have been released based on these [1,2,3,4,5]. One of the representative technical methods for measuring glucose is an electrochemical method based on glucose oxidase (GOD). The reaction occurring in the GOD enzyme layer can be expressed as follows:

glucose + O_2_ → gluconolactone + H_2_O_2_ (1)

In the above formula, glucose can be measured indirectly by electrically measuring the amount of oxygen consumed or the amount of hydrogen peroxide produced in the GOD reaction. However, if we use a Clark electrode, which is commonly used to measure oxygen, we inevitably consume oxygen for the measurement. Therefore, under conditions where the available oxygen in the sample is limited, the above GOD reaction may not be sufficient, and thus the measured glucose value may be distorted [5]. In addition, for continuous glucose monitoring (CGM), which has recently become more important, it is necessary to attach a blood glucose sensor to the skin for a certain period. The electrochemical glucose sensor in the implanted state poses problems, such as poor accuracy [6]. Applying electrical energy through an electrode to measure oxygen or hydrogen peroxide while the sensor is implanted can be a potential hazard affecting the human body [7,8].

In order to solve these problems, glucose measurement technology based on glucose oxidation and optical oxygen measurement has been drawing attention [9,10,11,12,13,14,15]. This has the form of a multi-film in which a substance capable of optically measuring oxygen (such as ruthenium, Ru) is fixed to a matrix and a GOD layer is laminated thereon. Materials used as the matrix include nylon, polyimide, and sol-gel. However, these sensors require a complex process, such as plasma treatment on a glass substrate, in order to improve the adhesion of the oxygen sensing film.

Pressure sensitive paint (PSP) is widely used to measure fluid dynamic pressure and velocity in a two-dimensional manner in a specific lighting and image acquisition condition by painting on the surface of a subject, such as a model of an airplane or automobile [16,17]. This is based on the quenching of luminescent compound (usually Ru) dispersed in the paint, depending on the presence or absence of surrounding oxygen molecules. In the excitation condition of a specific wavelength, light emission of light-emitting molecules decreases when the partial pressure of oxygen is high, and the degree of light emission increases when the partial pressure is low. As such, it is possible to measure the partial pressure of oxygen using a pressure sensitive paint, so in the early stages of this study, it was expected that glucose could be measured indirectly by combining it with a GOD layer. PSP has already been commercialized and sold in the form of a spray can. It has excellent adhesion to the surface of various materials without special physical or chemical treatment, and it is easy to control the film thickness. Therefore, it was expected that it would be possible to manufacture an optical glucose sensor with superior properties more simply than by the conventional method.

This study aimed to develop an optical glucose sensor film that could be fabricated by a simple process using commercial PSP. The glucose sensor manufacturing technology using PSP presented in this study was greatly simplified compared with the conventional electrochemical or optical glucose sensor production process. In addition, a glucose concentration analysis method was developed using the image of the sensor acquired with a color image sensor. Through the developed sensor and analysis technology, a basis for glucose measurement was established that enables two-dimensional, online, and continuous measurement. Detailed steps performed to achieve these research goals were as follows:

(1) Design and fabrication of glucose sensor film using PSP;
(2) Spectrum analysis of the output optical signal of the manufactured glucose sensor by spectrometry;
(3) Development of a quantitative analysis method through color image analysis (photometry) of glucose sensor;
(4) Comparison of glucose quantification methods.

## 2. Materials and Methods

### 2.1. Glucose Sensor Manufacturing

#### 2.1.1. Commercial PSP for Glucose Sensor

As explained earlier, the PSP is originally a paint for fluid dynamic pressure measurement. For the manufacture of a glucose sensor using PSP in this study, a commercially available single-luminophore paint packaged in an aerosol can (UniCoat PSP, product ID: UNC-12, Innovative Scientific Solutions, Inc., Dayton, OH, USA) was used for easy thickness control. According to the manufacturer’s specifications, the temperature sensitivity of the selected PSP is 1.3% per degree Celsius, the response time is 750 ms, and the excitation and emission are 380 to 520 nm and 620 to 750 nm, respectively [18]. Since the photo-degradation rate due to light excitation is mentioned as 1% per hour, it was necessary to store the sensor in a light-blocked container after manufacturing.

#### 2.1.2. Glucose Oxidase Enzyme Layer

The proposed glucose sensor is based on the optical detection of oxygen consumed by the GOD-catalyzed oxidation of β-d-glucose [19]. It uses glutaraldehyde (GA) to crosslink GOD with bovine serum albumin (BSA). β-d(+) glucose (EC 207-756-2), GOD (EC 1.1.3.4, 15,500 units/g), BSA (EC 232-936-2), and GA (EC 203-856-5) were obtained from Sigma (Sigma-Aldrich Co., St. Louis, MO, USA). In general, a thicker enzyme layer shows better linearity in a wider concentration range, but the response time is longer. A smaller ratio of glutaraldehyde may result in insufficient immobilization, while a higher ratio may reduce the efficiency of measurement due to more immobilization than necessary. The glucose enzyme solution included 0.75 mg of BSA and 0.75 mg of GOD in a 15 μL of 10 mM phosphate buffer solution. To promote adhesion between the enzyme membrane and the PSP surface, 20 μL of 1 wt% 3-aminopropyltriethoxysilane (3-APTES) was applied and cured for 30 min at 80 °C for surface silanization. Next, 15 μL of the enzyme solution was cast on the silanized area with a microsyringe. Then, 15 μL of 5 wt% GA was applied to initiate the chemical crosslinking reaction of BSA on the PSP surface. All other chemicals were of analytical reagent grade. Deionized water was used throughout the experiments for the preparation of samples, buffers, and other solutions.

#### 2.1.3. Structure of the Glucose Sensor

**Figure 1:**
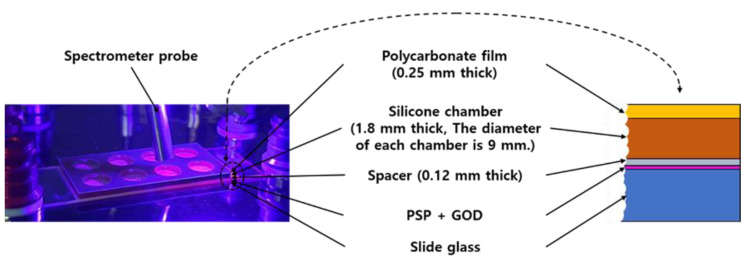
Photo (left) and cross-sectional view (right) of the produced glucose sensor. The photo also shows the spectrometer probe used for the measurement.

The sensor manufactured in this study had a structure as shown in the Figure 1. First, PSP was sprayed on the surface of the slide glass. With one spray, an 80 μm thick PSP layer was obtained, and 3 sprays were performed to finally create an oxygen-sensitive layer with a thickness of 240 μm. After 24 h of drying, a spacer (SKU654008, Grace Bio-Labs, Bend, OR, USA) was attached to the PSP surface. There were 8 holes in the spacer, and the diameter of each hole was 9 mm. The hole of the spacer served as a kind of chamber for GOD casting. After finishing the GOD casting and crosslinking for 24 h, a silicone chamber (SKU621201, Grace Bio-Labs, Bend, OR, USA) was attached, as shown in the Figure 1, and it was equipped with a polycarbonate film cover with holes for the entrance and exit of the sample solution, using a pipette at the top.

### 2.2. Glucose Quantification

#### 2.2.1. Measurement Fixture

**Figure 2:**
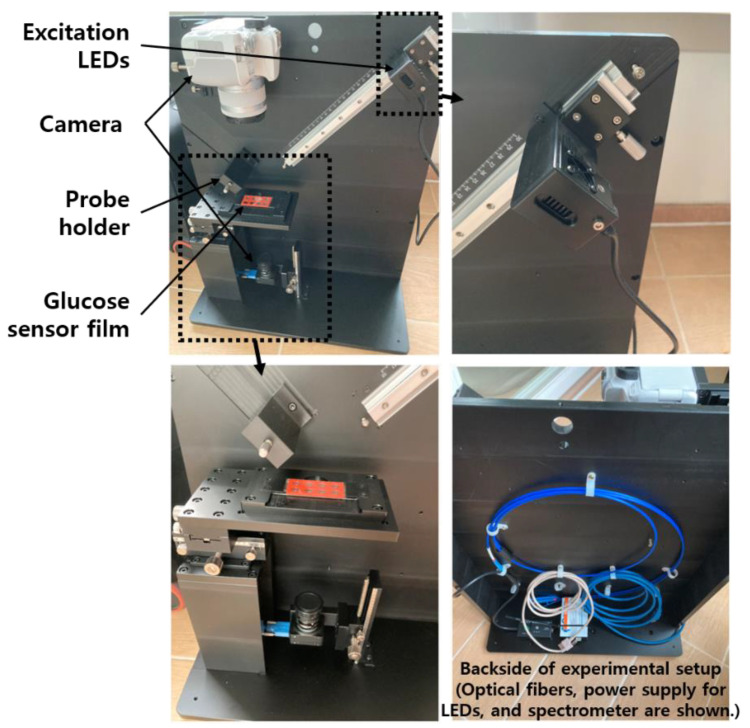
Photos of a test fixture for quantitative analysis of a prototype glucose sensor.

The center wavelengths of excitation light and emission light given in the specifications of the PSP used in this study were 420 and 655 nm, respectively [18]. When a GOD layer for glucose measurement is added to a PSP having these characteristics, the central wavelength of the emitted light may be slightly changed. However, as this can only be known through measurement, the necessary measurement fixture was designed based on the optical characteristics of PSP only. For this, the necessary excitation light source, charge-coupled device (CCD) camera, optical filters, and spectrometer were selected, and a measuring fixture was designed. Its photos are shown in the Figure 2. A general digital single-lens reflex (DSLR) camera and a CCD camera for microscopes were mounted on the upper and lower sides of the glucose sensor, respectively, and the distance between each camera and the subject (i.e., glucose sensor) was adjustable. The LED excitation light source and the spectrometer probe could be adjusted in distance to the glucose sensor, and each centerline was at 45 degrees to the vertical line of the sensor film plane. During the measurement, the entire fixture was covered with a black cloth, or all other lights around it were turned off to create an optically separated environment.

#### 2.2.2. Spectrometric and Photometric Measurements

A spectrometer and probe were used as the first glucose quantification method in this research. This method uses a spectrometer to measure the intensity of light emission, and only the signal in the wavelength band responding to the change in glucose concentration is used for quantification. Thus, even if the area of the produced glucose sensor film is wide, it is quantified through only a part of the light emission.

The second measurement was performed by acquiring an image of the glucose sensor, extracting the intensity of each pixel in the region of interest (ROI) and using it for glucose quantification. This method allows quantification of the entire sensor film with a single image, so when applying the developed sensor film to a situation such as cell culture, it is possible to obtain a two-dimensional image of chemical concentration through a simple process.

**Figure 3:**
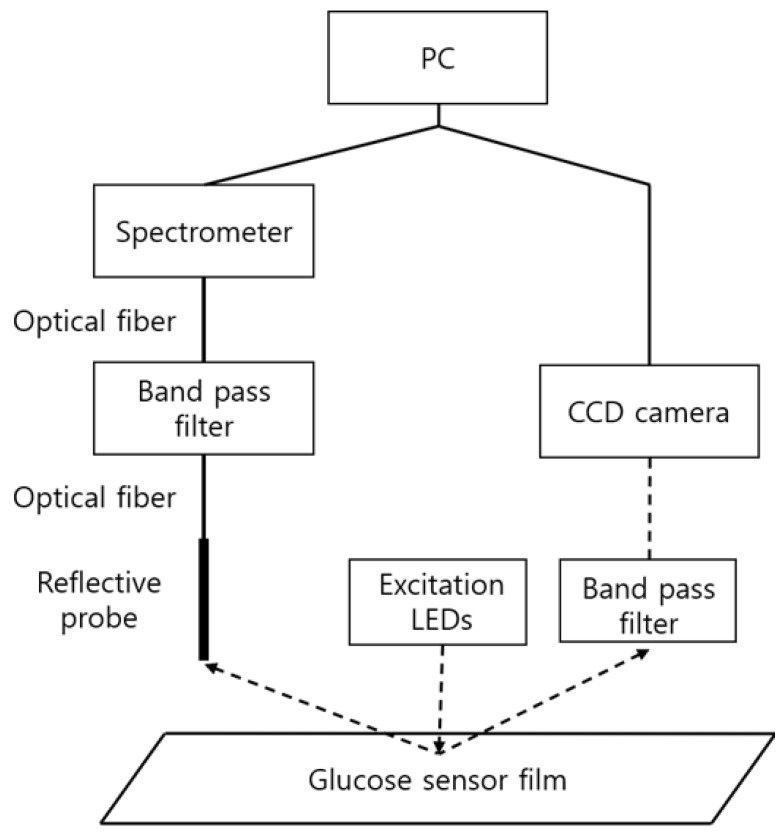
Measurement components for spectrometry and photometry. The left side shows the connection diagram of the components for spectrometry and the right side the photometry connection diagram.

The above methods are referred to as spectrometry and photometry, respectively, and the required components and the diagram are as shown in Figure 3. Excitation LEDs (Prime-100, 405 nm, Skycares, Gimpo, Korea) were commonly used for both measurement methods. First, in spectrometry, the emitted light from the glucose sensor film was collected through a reflective probe (R400-7-UV-VIS, Ocean Insight, Orlando, FL, USA) and measured in a spectrometer (USB2000, Ocean Insight, Orlando, FL, USA) through an optical fiber and a band pass filter (OF2-OG515, Ocean Insight, Orlando, FL, USA). On the other hand, in photometry, the light emitted from the sensor passed through a band pass filter (BN650, Midwest Optical Systems, Inc., Palatine, IL, USA), and then acquired through a camera (CM3-U3-13S2C-CS FLIR Systems, Wilsonville, OR, USA) equipped with a lens with an appropriate magnification (M1214-MP, Computar, Cary, NC, USA).

## 3. Results and Discussion

### 3.1. Spectrum Analysis of the Output Optical Signal of the Glucose Sensor: Spectrometry

**Figure 4:**
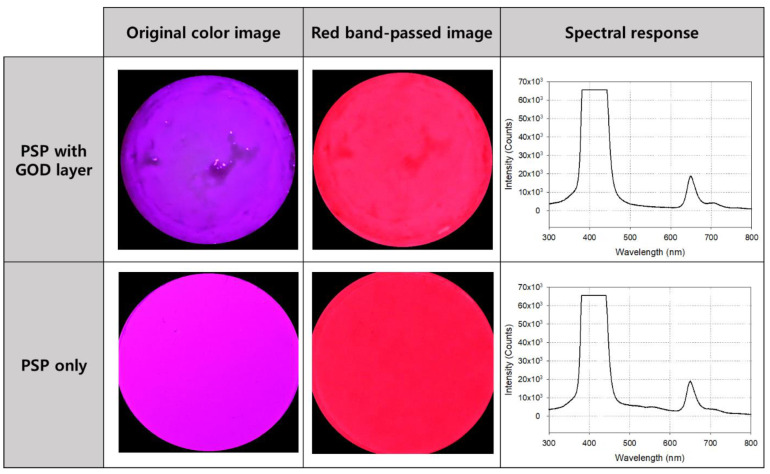
Photos and the excitation and emission spectrums of the sensor before (lower row) and after (upper row) the glucose oxidase (GOD) layer application. The spectral curves on the right side were obtained without using a bandpass filter.

The photos and spectrum measurement results of the oxygen sensor coated only with PSP and the glucose sensor with the GOD layer added to the PSP are shown in Figure 4. The first picture in the lower row is an image taken after PSP coating without a band pass filter, and the second picture is the image that has passed through the filter. Its spectrum is shown on the right side of the row, and the central wavelength and maximum intensity of the emitted light can be identified on it. The upper row shows the sensor images (before and after using the filter) and their spectral characteristics after laminating the GOD layer on the PSP. As shown in each spectral curve, there was no significant difference between the center wavelength of emission light when only PSP was applied and when GOD was stacked on the PSP. Therefore, the structure and parts of the measuring fixture configured to measure the emitted light of the PSP could be used as they are for the measurement using the glucose sensor.

From the basic experimental data shown in Figure 4, it was predicted that there would be a change in intensity in the 600–700 nm wavelength band due to the difference in glucose concentration in the sample. The higher the glucose concentration, the more oxygen consumption due to the GOD reaction increases, and the surrounding oxygen concentration decreases. Accordingly, the quenching of luminescent molecules decreases and the intensity of light emission increases. In other words, the sensor becomes brighter.

In order to observe and quantify this more efficiently, an optical filter that passed light in the signal band and removed the remaining noise was applied. The selected bandpass filter with a central wavelength of 650 nm was used not only for spectrometry using a spectrometer probe, but also for glucose quantification using a color camera (i.e., photometry).

**Figure 5:**
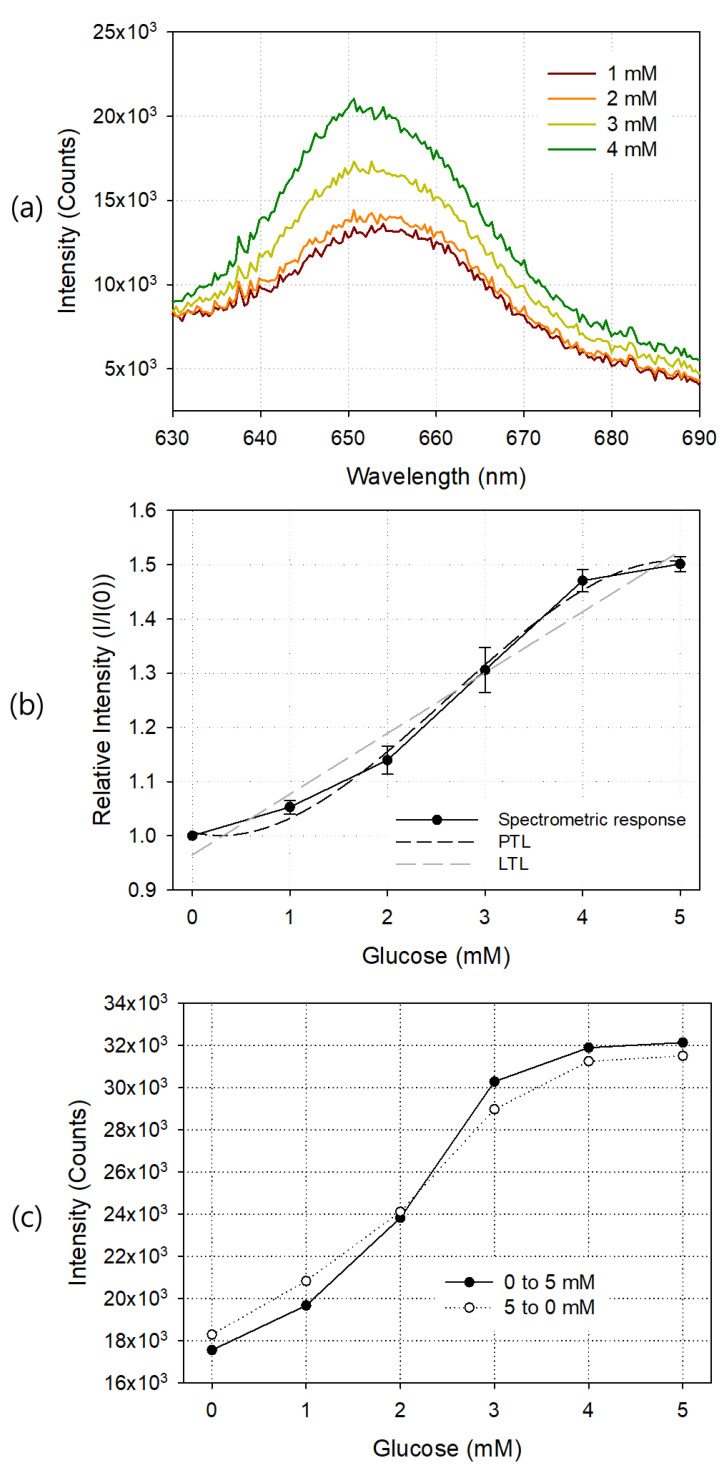
Exemplary output waveforms at 1, 2, 3, and 4 mM glucose (**a**), spectrometric responses (**b**), and hysteresis curve (**c**) of the manufactured glucose sensor. In (**b**), linear trendline (LTL) and third degree polynomial trendline (PTL) for the response curve are also shown. Each result is an average of five measurements, and the error bar represents the standard deviation.

Using the prepared glucose sensor, measuring fixture, and spectrometer, the change of the sensor output optical signal according to the concentration of glucose was observed in terms of wavelength, and the results are shown in Figure 5. Sample solutions with glucose concentrations of 0, 1, 2, 3, 4, 5, 10, 15, and 20 mM were sequentially injected, and each sensor response was measured. Figure 5a shows the output waveforms around the 650 nm wavelength at 1, 2, 3 and 4 mM of glucose. As the glucose concentration of the sample solution increased, the output light intensity increased. Since the diameter of the silicon circular chamber used to fabricate the glucose sensor was 9 mm and the height was 1.8 mm, the volume of the glucose sample solution to be injected once was about 115 μL. In each spectrum of the glucose sensor, the average value of the light intensity in the wavelength range of 640 to 660 nm was calculated, and these values were plotted according to the glucose concentration as shown in Figure 5b. I and I(0) represent the average emission intensity with and without glucose, respectively. The fabricated glucose sensor showed good linearity at 0–4 mM, and the light output was saturated at concentrations exceeding 5 mM. The limit of detection (LOD) of the fabricated glucose sensor was 0.37 mM, and as the concentration of GOD increased, the LOD value became smaller, but the linear detection range also tended to be reduced. According to the manufacturer’s specification, the response time of PSP alone is only 750 ms, but the GOD reaction required for glucose measurement took more time, and the change in light output according to the change in glucose concentration was stabilized after about 2 min. Table 1 shows the comparison of the performance of the glucose sensor we developed with those of other authors. In all cases, including this study, response time and linear range varied depending on the concentration of the GOD enzyme and the amount of oxygen available in the sample. The dark and lighter dotted lines in the graph show the third-degree polynomial trendline (PTL) and linear trendline (LTL) of the measured values, respectively. The LTL of the spectrometric output curve is y=0.1122x+0.9646 and $R^{2}=0.9537$. The third-degree PTL is $y=-0.0100x^{3}+0.0787x^{2}-0.0431x+1.0067$ and $R^{2}=0.9839$.

Figure 5c shows the hysteresis curve of the glucose sensor. A glucose sample solution up to 5 mM was sequentially injected, starting at 0 mM and increasing by 1 mM, into the silicon chamber, and the spectrometric response of each injection was observed. Next, starting at 5 mM and decreasing the concentration to 0 mM, the output was observed. Since the output values between concentrations were sufficiently distinguishable from each other, we concluded that they had good hysteresis characteristics.

### 3.2. Image Analysis of the Glucose Sensor: Photometry

**Figure 6:**
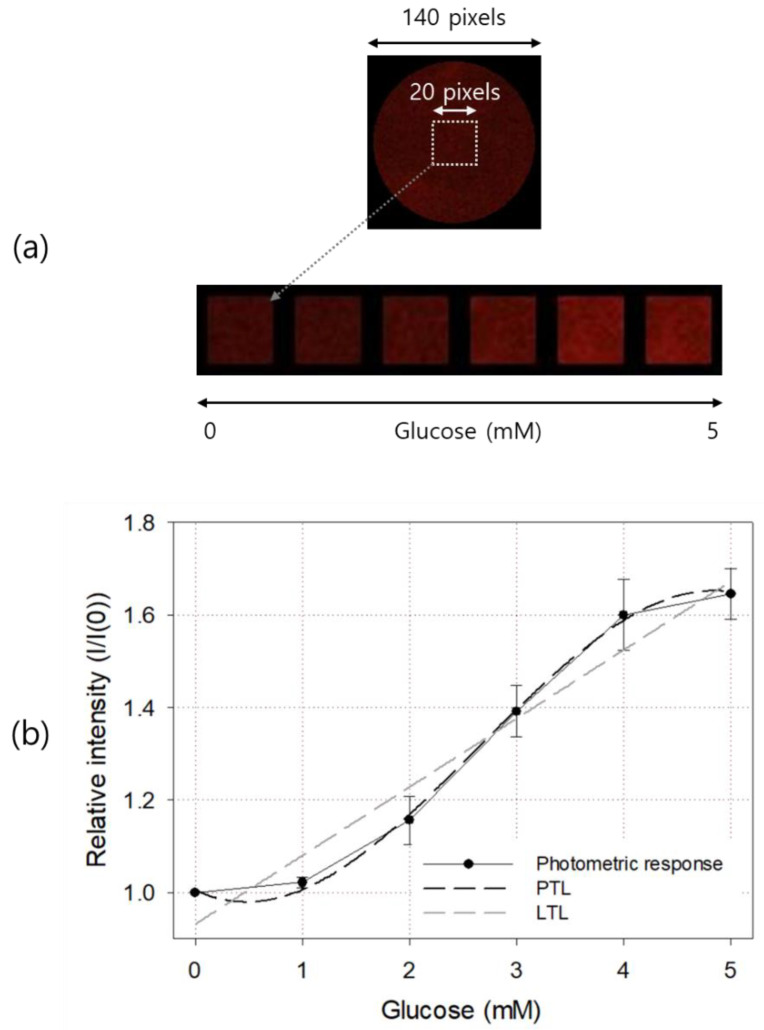
Color images (**a**) and photometric responses (**b**) of the manufactured glucose sensor. In (**b**), LTL and third-degree PTL for the response curve are also shown. The result is an average of five measurements, and the error bar represents the standard deviation.

The top picture in Figure 6a is a glucose sensor with a diameter of 9 mm and the emitted light from it was taken using a color CCD camera and an optical band-pass filter. It was originally taken to have a size of 140 × 140 pixels. A 20 × 20 pixels square ROI concentric with the sensor center was designated, and its average pixel intensity was calculated and used to determine the glucose concentration of the sample solution. The bottom pictures in Figure 6a are images of sensor ROIs taken after injecting 0, 1, 2, 3, 4, and 5 mM glucose solutions. As shown in the figure, the difference in brightness between each ROI could be recognized even with the naked eye.

The result of quantification based on the image of the glucose sensor is shown in Figure 6b. The basic response curve shape and linear range were similar to those obtained by spectrometry. Before measurement, a glucose-free (i.e., air-saturated) sample was injected and the brightness of the excitation light and camera shutter time were adjusted so that the pixel intensity of the 8-bit image sensor was about 1/3 of the maximum brightness of 255 counts. In glucose above 4 mM, the excitation light of the sensor was less than 2/3 of the maximum pixel brightness, so we inferred that the nonlinear trend above 4 mM was not caused by the optical measurement equipment. Similar linear characteristics were also observed in cases of other optical glucose sensors based on the amount of oxygen consumed in the GOD enzyme layer [12,13,14,15], so this seemed to be attributable to the characteristics of Ru quenching or the GOD enzymatic reaction [9]. The PTL and LTL of the obtained response curve are shown in the figure as a dark dotted line and a light dotted line, respectively. The LTL of the photometric output curve is y=0.1484x+0.9313 and $R^{2}=0.9283$. The third-degree PTL is $y=-0.0163x^{3}+0.1299x^{2}-0.1123x+1.0049\;$ and $R^{2}=0.9749$.

### 3.3. Comparison of Quantitication Results of Each Analysis Method

**Figure 7:**
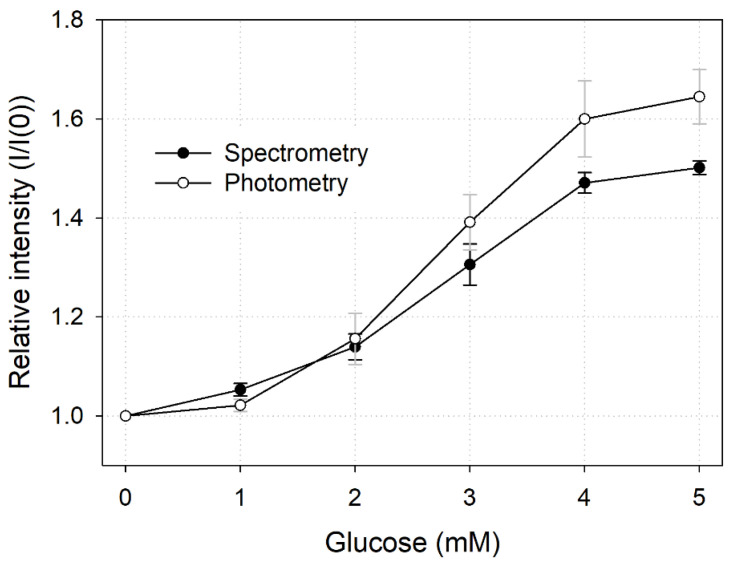
Comparison of spectral and photometric glucose quantification methods.

To compare the results of the measurement methods described above (i.e., spectrometry and photometry), we plotted them in one graph (Figure 7). As the slopes of spectrometry LTL and photometry LTL were 0.1122 and 0.1484, respectively, we concluded that the sensitivity of photometry was relatively higher. However, the standard deviation of each measurement value of photometry was large, so the precision of the sensor was inferior to that of spectrometry. In conclusion, photometry was a relatively simple and convenient measurement method as it made it possible to quantify glucose in multiple points with only a single image, but there was a slight decrease in performance compared with the measurement method using a spectrometer.

## 4. Conclusions

This study aimed at the development of an optical glucose sensor with a simple manufacturing process. This sensor is composed of a layer that responds to the partial pressure (i.e., concentration) of oxygen due to the applied commercially available PSP, and it measures the amount of glucose thanks to the GOD enzyme fixed on it in an appropriate way. The glucose sensor we fabricated showed good linearity at 0–4 mM, and the LOD was 0.37 mM. In addition, it was possible to quantify or visualize the glucose concentration in two dimensions only by analyzing the color image of the sensor without complex and expensive equipment. If this glucose sensor film is applied to glucose measurement in a cell culture, real-time continuous two-dimensional measurement of glucose will be possible.

## Figures and Tables

**Table 1:** Comparison of response time and linear analytical range with existing research cases.

Source	Response Time	Linear Analytical Range
This research	2 min.	0.37–4 mM
Reference [12]	2 min.	0–5 mM ^1^
Reference [13]	8–60 s.	0.01–2 mM
Reference [14]	6 min.	0.06–1 mM
Reference [15]	9–28 s.	0.6–20 mM ^2^

^1^ Limit of detection is unknown. ^2^ The upper limit of the linear range varied from 5 to 20 mM, depending on the partial pressure of oxygen in the sample.

## Data Availability

Not applicable.
